# Chromium picolinate therapy in pre/diabetes care: recognition of individual risks and outcomes

**DOI:** 10.1186/1878-5085-5-S1-A77

**Published:** 2014-02-11

**Authors:** Olga Golubnitschaja, Kristina Yeghiazaryan, Hans H Schild

**Affiliations:** 1 Radiologic Clinic, University of Bonn, Germany

## Scientific background

Due to the important physiologic function of trivalent chromium in glucose/insulin homeostasis, some commercial organisations promote Cr3^+^ supplements in maintaining proper carbohydrate and lipid metabolism; regulation of reducing carbohydrate carvings and appetite; prevention of insulin resistance and glucose intolerance; regulation of body composition, including reducing fat mass and increasing lean body mass; optimal body building for athletes; losing weight; treatment of atypical depression as an antidepressant; and prevention of obesity and type 2 diabetes mellitus. On one hand, case reports are commented as ‘nonevidence-based anecdotes’. On the other hand, a number of independent studies warn against adverse health outcomes assigned to chromium picolinate (CrPic) dietary application [1].

## Materials and methods

To simulate treatment affects by CrPic a well-acknowledged animal diabetes model of db/db-mice was utilised. Animals were grouped into eight groups. Group 1 represented the healthy control. Group 2 was the model mice for type 2 DM. Groups 3 - 7 were diabetes-model mice treated under individual algorithms: 5 mg/kg CrPic from 6 weeks to 6 months of age (Group 3), 10 mg/kg CrPic from 6 weeks to 6 months of age (Group 4), 100 mg/kg CrPic from 6 weeks to 6 months of age (Group 5), 100 mg/kg CrPic from 3 months to 6 months of age (Group 6), and 250 mg/kg CrPic from 3 months to 6 months of age (Group 7). Kidney tissue samples were collected and stored at - 80°C till quantitative comet assay sub-cellular imaging was performed to evaluate individual and group-specific effects by CrPic treatments.

## Results

**Figure 1:**
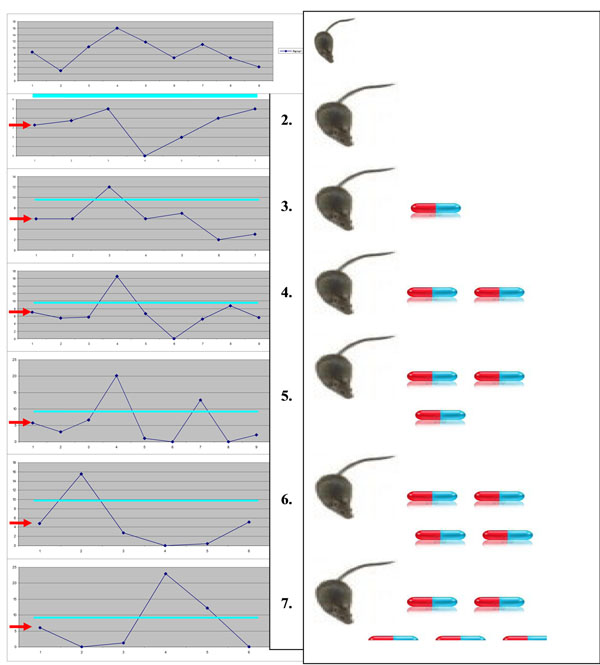
Diagram demonstrates individual reactions towards CrPic treatments in the groups of comparison. Individual levels of intact DNA (axe X, individual numbering; axe Y, comet class I in %) are highly heterogeneous in diabetic groups compared to control group 1. The highest level of heterogeneity is evident in groups 5, 6 and 7 with the highest doses of CrPic supplements. The turquoise lines mark the level corresponding to the mean value of the control group; in contrast, the red arrows mark the level corresponding to the mean of the untreated diabetic group [2].

We have demonstrated highly individual reactions towards CrPic dietary supplements and highlighted risks when the dietary supplements are used freely as therapeutic agents, without application of advanced diagnostic tools to predict individual outcomes (Figure 1) [2].

## Conclusions and recommendations: artificial supplements for diabetes prevention - hype or hope?

High efficacy of a balanced diet, an individually optimised lifestyle and personalised treatment regiments can hardly be substituted by a limited number of single supplements to cover all the multifactorial risks such as the upward trends of population ageing, environmental risk factors, urbanisation, additive effects of diverse stress factors, incorrectly chosen lifestyle including unfavourable nutritional habits, increasing prevalence of obesity, low physical activity, etc [3]. The population at-risk for diabetes is huge and increasing in a pandemic scale. One of the reasons might be the failed attempt to prevent the disease by the application of artificial supplements and drugs with hardly recognised individual risks. Consequently, a multimodal approach of integrative medicine by predictive diagnostics, targeted prevention and individually created treatment algorithms is highly desirable. Targeted measures require a creation of new guidelines that are essential to regulate (renoprotective) therapy approaches and the application of more individualised therapeutic modalities for advanced diabetes care. These measures should provide a legitimate regulation for well-timed predictive diagnostics, an effective prevention and the creation of individualised treatment algorithms in pre/diabetes care [4].
